# Characterization of Chicken Thrombocyte Responses to Toll-Like Receptor Ligands

**DOI:** 10.1371/journal.pone.0043381

**Published:** 2012-08-20

**Authors:** Michael St. Paul, Sarah Paolucci, Neda Barjesteh, R. Darren Wood, Karel A. Schat, Shayan Sharif

**Affiliations:** 1 Department of Pathobiology, Ontario Veterinary College, University of Guelph, Guelph, Ontario, Canada; 2 Department of Microbiology and Immunology, College of Veterinary Medicine, Cornell University, Ithaca, New York, United States of America; University of California, Davis, United States of America

## Abstract

Thrombocytes are the avian equivalent to mammalian platelets. In addition to their hemostatic effects, mammalian platelets rely in part on pattern recognition receptors, such as the Toll-like receptors (TLR), to detect the presence of pathogens and signal the release of certain cytokines. Ligands for TLRs include lipopolysaccharide (LPS), which is bound by TLR4, as well as unmethylated CpG DNA motifs, which are bound by TLR9 in mammals and TLR21 in chickens. Similar to mammalian platelets, avian thrombocytes have been shown to express TLR4 and secrete some pro-inflammatory cytokines in response to LPS treatment. However, the full extent of the contributions made by thrombocytes to host immunity has yet to be elucidated. Importantly, the mechanisms by which TLR stimulation may modulate thrombocyte effector functions have not been well characterized. As such, the objective of the present study was to gain further insight into the immunological role of thrombocytes by analyzing their responses to treatment with ligands for TLR4 and TLR21. To this end, we quantified the relative expression of several immune system genes at 1, 3, 8 and 18 hours post-treatment using real-time RT-PCR. Furthermore, production of nitric oxide and phagocytic activity of thrombocytes was measured after their activation with TLR ligands. We found that thrombocytes constitutively express transcripts for both pro- and anti-inflammatory cytokines, in addition to those associated with anti-viral responses and antigen presentation. Moreover, we found that both LPS and CpG oligodeoxynucleotides (ODN) induced robust pro-inflammatory responses in thrombocytes, as characterized by more than 100 fold increase in interleukin (IL)-1β, IL-6 and IL-8 transcripts, while only LPS enhanced nitric oxide production and phagocytic capabilities. Future studies may be aimed at examining the responses of thrombocytes to other TLR ligands.

## Introduction

Thrombocytes are the most abundant leukocyte found in the blood of chickens [1]. Functioning in a manner similar to that of mammalian platelets, thrombocytes promote hemostasis in response to vascular injury by aggregating and releasing prothrombotic factors [2], [3]. Furthermore, similar to mammalian platelets, evidence suggests that chicken thrombocytes may have an immunological role as well [4], [5]. In mammals, it has become increasingly clear that platelets secrete anti-microbial molecules and cytokines that modulate inflammatory responses [6]. Examples of such molecules include the pro-inflammatory cytokine interleukin (IL)-1β, as well as the anti-inflammatory cytokine transforming growth factor (TGF)-β [7], [8]. Platelets store these cytokines in granules and release them upon sensing danger, relying in part on Toll-like receptors (TLRs) to detect the presence of pathogens and initiate this process [6], [9]. Being a pattern recognition receptor, TLRs bind conserved structural motifs of pathogens termed pathogen associated molecular patterns (PAMPs). Ligands for TLRs include lipopolysaccharide (LPS), which is bound by TLR4, as well as unmethylated CpG DNA motifs found in the nucleic acids of certain bacteria and viruses, which are bound by TLR9 in mammals and TLR21 in chickens [10], [11].

It has been reported that both mouse and human platelets express TLRs 1–9, while only the expression of TLR4 has been detected in chicken thrombocytes so far [12]. Treating thrombocytes with LPS promotes the up-regulation of transcripts for the cytokines IL-1β, IL-6 and IL-12 in addition to transcripts for cyclooxygenase-2 and prostaglandin E2, thereby suggestive of a potential role of thrombocytes in the inflammatory response [5], [12]. However, the full extent of the contributions made by thrombocytes to host immunity has yet to be elucidated. Importantly, the mechanisms in which TLR stimulation may modulate cytokine production and thrombocyte effector functions have not been well characterized. The objective of the present study was to gain further insight into the immunological role of thrombocytes by analyzing their responses to treatment with ligands for TLR4 and TLR21. We found that thrombocytes constitutively express transcripts for several TLRs in addition to genes associated with inflammation, antigen presentation and anti-viral responses. Moreover, treatment with the TLR ligands LPS and CpG ODN significantly up-regulated many of these genes, while only LPS enhanced nitric oxide production and phagocytic capabilities.

## Materials and Methods

### Chickens

Six-week-old broiler chickens were procured from the Arkell Poultry Research Center, University of Guelph (Guelph, ON). This research was approved by the University of Guelph Animal Care Committee and complied with the guidelines of the Canadian Council on Animal Care.

### TLR Ligands

LPS from *Escherichia coli* 0111:B4 was purchased from Sigma-Aldrich-Canada (Oakville, ON), while synthetic class B CpG ODN 2007 [5′- TCGTCGTTGTCGTTTTGTCGTT-3′] and non-CpG ODN [5′- TGCTGCTTGTGCTTTTGTGCTT-3′] were purchased from Eurofins MWG Operon (Ebersberg, GER). All of the ligands used were re-suspended in sterile phosphate buffered saline (PBS, pH 7.4) and diluted to working concentrations in complete RPMI medium.

### Thrombocyte Isolation and Stimulation

Thrombocytes were isolated as previously described [12]. Briefly, blood was collected from the wing vein of chickens and diluted 1∶1 in Alsever’s solution. The diluted blood was overlaid onto a Histopaque-1077 (Sigma-Aldrich, Oakville, ON) gradient and centrifuged at 1700 g for 30 minutes, and the thrombocytes were harvested from the plasma-Histopaque interface and washed 3x in RPMI-1640 (Invitrogen, Burlington, ON) supplemented with 10% heat-inactivated fetal bovine serum, 200 U/mL penicillin, 80 µg/mL streptomycin, 25 mg gentamicin, 10 mM HEPES buffer, 50 µM β-mercaptoethanol**,** and 2 mM L-glutamine**.** Cell purity was assessed by Wright-Giemsa staining. Thrombocytes from 5 individual chickens were seeded into 48-well plates at 1×10^7^ cells/mL for *in vitro* stimulation with either a low (1 µg/mL) or high (5 µg/mL) dose of each TLR ligand, while the control groups received non-CpG ODN (5 µg/mL) or medium. Cells were cultured at 41°C with 5% CO_2_ and harvested at 1, 3, 8 and 18 hours post-stimulation for RNA extraction. Supernatants were collected at 48 hours post-treatment for nitrite determination.

### RNA Extraction and cDNA Synthesis

Total RNA was extracted from thrombocytes using TRIzol® (Invitrogen, Carlsbad, CA) according to the manufacturer’s protocol and treated with DNA Free® (Ambion, Austin, TX) DNAse. Subsequently, 500 ng of purified RNA was reverse transcribed to cDNA using Superscript® II First Strand Synthesis kit (Invitrogen, Carlsbad, CA) and oligo-dT primers, according to the manufacturer’s recommended protocol. The resulting cDNA was subsequently diluted 1∶10 in DEPC treated water.

### Real-time PCR

Quantitative real-time PCR using SYBR Green was performed on diluted cDNA using the LightCycler® 480 II (Roche Diagnostics GmbH, Mannheim, GER) as previously described [13]. Briefly, each reaction involved a pre-incubation at 95°C for 10 min, followed by 45 cycles of 95°C for 10 min, 55°C–64°C (T_A_ as per primer) for 5 s, and elongation at 72°C for 10 s. Subsequent melt curve analysis was performed by heating to 95°C for 10 s, cooling to 65°C for 1 min, and heating to 97°C. Primers were synthesized by Sigma-Aldrich-Canada (Oakville, ON), and their specific sequences and accession numbers are outlined in Table 1. Relative expression levels of all genes was calculated relative to the housekeeping gene β-actin using the LightCycler® 480 Software (Roche Diagnostics GmbH, Mannheim, GER), based on the formula developed by Pfaffl [14]. Data represent mean of 5 biological replicates (chickens) ± standard error.

**Table 1:** Primer sequences and accession numbers used for real-time PCR.

Target Gene	Primer Sequence	GenBank Accession Number
IL-1β	F: 5′- GTGAGGCTCAACATTGCGCTGTA -3′	Y15006
	R: 5′- TGTCCAGGCGGTAGAAGATGAAG -3′	
IL-6	F: 5′- CGTGTGCGAGAACAGCATGGAGA -3′	NM_204628.1
	R: 5′- TCAGGCATTTCTCCTCGTCGAAGC -3′	
IL-8	F: 5′- CCAAGCACACCTCTCTTCCA -3′	AJ009800
	R: 5′- GCAAGGTAGGACGCTGGTAA -3′	
IL-10	F: 5′- AGCAGATCAAGGAGACGTTC -3′	AJ621614
	R: 5′- ATCAGCAGGTACTCCTCGAT -3	
IL-12p40	F: 5′- CCAAGACCTGGAGCACACCGAAG -3′	AY262752.1
	R: 5′- CGATCCCTGGCCTGCACAGAGA-3′	
IFN-α	F: 5′- ATCCTGCTGCTCACGCTCCTTCT -3′	AB021154
	R: 5′- GGTGTTGCTGGTGTCCAGGATG -3′	
IFN-β	F: 5′- GCCTCCAGCTCCTTCAGAATACG -3′	AY974089
	R: 5′- CTGGATCTGGTTGAGGAGGCTGT -3′	
TGF-β	F: 5′- CGGCCGACGATGAGTGGCTC-3′	M31160.1
	R: 5′-CGGGGCCCATCTCACAGGGA-3′	
CD40	F: 5′- CCTGGTGATGCTGTGAATTG -3′	AJ293700
	R: 5′- CTTCTGTGTCGTTGCATTCAG -3′	
CD80	F: 5′- CTGTTCCTTCACATCCTGAGAG -3′	Y08823
	R: 5′- CTTCAACACCATCTATTTGCCAG -3′	
MHC-II	F: 5′- CCACGGACGTGATGCAGAAC -3′	113206149
	R: 5′- ACCGCGCAGGAACACGAAGA -3′	
iNOS	F: 5′- GGCAGCAGCGTCTCTATGACTTG -3′	NM 204961
	R: 5′- GACTTTAGGCTGCCCAGGTTG -3′	
OAS	F: 5′- AGAACTGCAGAAGAACTTTGTC -3′	AB002586
	R: 5′- GCTTCAACATCTCCTTGTACC -3′	
TLR2	F: 5′- ATCCTGCTGGAGCCCATTCAGAG -3′	NM_204278.1/NM_001161650
	R: 5′- TTGCTCTTCATCAGGAGGCCACTC -3′	
TLR3	F: 5′- TCAGTACATTTGTAACACCCCGCC -3′	DQ780341
	R: 5′- GGCGTCATAATCAAACACTCC -3′	
TLR4	F: 5′- TGCCATCCCAACCCAACCACAG -3′	AY064697.1
	R: 5′- ACACCCACTGAGCAGCACCAA -3′	
TLR5	F: 5′- TTCTTGCAACCTCACAGGTGTTCC -3′	NM_001024586.1
	R: 5′- CAGGTCCAAGACACGAAGATT -3′	
TLR7	F: 5′- TTCTGGCCACAGATGTGACC -3′	NM_001011688
	R: 5′- CCTTCAACTTGGCAGTGCAG -3′	
TLR21	F: 5′- CCTGCGCAAGTGTCCGCTCA -3′	AJ720600.1
	R: 5′- GCCCCAGGTCCAGGAAGCAG -3′	
β-Actin	F: 5′-CAACACAGTGCTGTCTGGTGGTA-3′	X00182
	R: 5′-ATCGTACTCCTGCTTGCTGATCC-3′	

### Nitrite and Phagocytosis Assays

Nitrite in culture supernatants was quantified using Griess reagent (Promega, Madison, WI) according to the manufacturer’s instructions. Phagocytosis was assessed using fluorescent latex beads (Cayman Chemical). Thrombocytes from four individual chickens were seeded into 96 well plates at 1×10^6^ cells/well and incubated with TLR ligands and latex beads for 2 hours. Medium was subsequently removed and background fluorescence was quenched with Trypan Blue. Fluorescence was assayed in a fluorescence plate meter (GloMax®- Multi, Promega). Freshly isolated chicken erythrocytes (1×10^6^ cells/well) treated with fluorescent latex beads were used as a non-phagocytic cell type control.

### Data Analysis

Statistical significance between treatment groups and the medium control group was calculated using a paired student’s t test and was considered statistically significant if p≤0.05 (*) and p≤0.01 (**), and in the case of CpG ODN, considered statistically significant from the non-CpG ODN control group if p≤ 0.05 (#).

## Results

### Thrombocytes Constitutively Express Several TLRs at the Transcript Level

**Figure 1:**
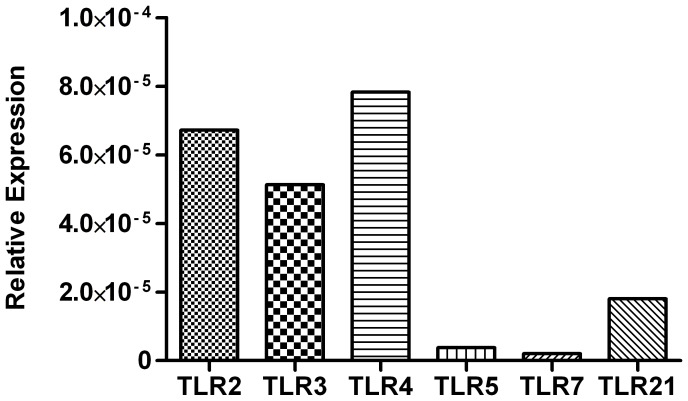
Relative gene expression of TLR transcripts in chicken thrombocytes. Gene expression of TLRs 2, 3, 4, 5, 7 and 21 transcripts in un-stimulated chicken thrombocytes relative to the house-keeping gene β-actin.

The presence and degree of expression of TLR transcripts have not yet been reported for chicken thrombocytes. To address this, we employed real-time PCR on un-stimulated thrombocytes to determine their repertoire of TLR expression (Figure 1). Our results suggest that thrombocytes constitutively express transcripts for TLRs 2, 3, 4, 5, 7 and 21. Furthermore, it appears that the transcripts for TLRs 2, 3 and 4 are the most abundant followed by transcripts for TLR21, while transcripts for TLRs 5 and 7 were the least abundant. Our results also suggest that thrombocytes express several cytokine and immune system genes at the transcript level (Table 2). We found that thrombocytes contain high levels of inducible nitric oxide synthase (iNOS), in addition to moderate levels of 2′–5′ oligoadenylate synthetase (OAS), IL-10 and the co-stimulatory molecule CD80. Lastly, we detected low levels of interferon (IFN)-β.

**Table 2:** Expression levels of gene transcripts in chicken thrombocytes.

Gene	Expression Level
iNOS	+++
2′–5′ OAS	++
IFN-β	+
CD80	++
IL-10	++

A “+++” indicates a high degree of expression, while a “++” indicates a moderate degree of expression, while a “+” indicates a low level of expression, as determined by real-time PCR.

### TLR Ligands Induce Robust Pro-inflammatory Responses

**Figure 2:**
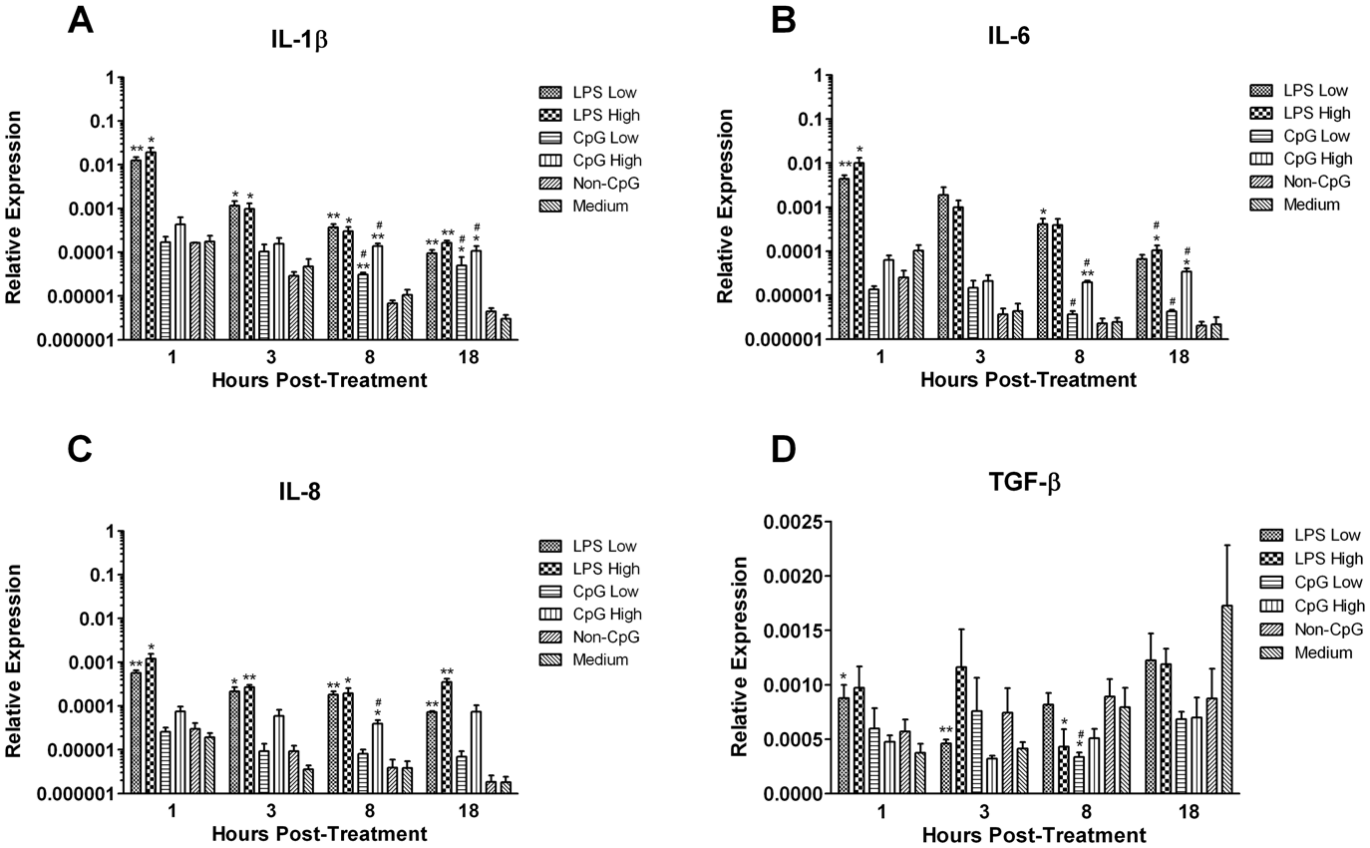
Relative gene expression of pro- and anti-inflammatory cytokines. Relative gene expression of the pro-inflammatory cytokines IL-1β, IL-6 and IL-8, and the anti-inflammatory cytokine TGF-β in chicken thrombocytes at 1, 3, 8 and 18 hours post-treatment with low (1 µg/mL) and high (5 µg/mL) doses of the TLR ligands LPS and CpG ODN. Data represent mean expression levels of target genes in 5 biological replicates relative to the house keeping gene β-actin ± standard error. Statistical significance between treatment groups and the medium control group was calculated using a paired student’s t test and was considered statistically significant if p≤0.05 (*) and p≤0.01 (**), and in the case of CpG ODN, considered statistically significant from the non-CpG ODN control group if p≤0.05 (#).

To elucidate the kinetics of the thrombocyte response following treatment with TLR ligands, we quantified transcripts for the pro-inflammatory cytokines IL-1β, IL-6 and IL-8, and the anti-inflammatory cytokine TGF-β (Figure 2). Treatment with either LPS or CpG ODN resulted in robust pro-inflammatory cytokine production, with approximately a 2 log-fold increase in IL-1β transcripts at several time points (Figure 2A). Responses peaked at 1 hour post-treatment (p≤0.05) and 8 hours post-treatment (p≤0.01) with high dose LPS and CpG ODN, respectively. A similar phenomenon was noted with respect to the expression of IL-6 in response to treatment with LPS, as a ∼2–3 log increase was observed throughout all of the sampling time points, while transcripts were up-regulated between 1–2 log in response to CpG ODN only at 8 and 18 hours post-treatment (Figure 2B). Similarly, for IL-8, transcripts were significantly up-regulated by ∼2 log in response to LPS throughout all of the sampling time points (Figure 2C). However, CpG ODN was not as efficacious as LPS, as IL-8 transcripts were up-regulated by ∼1 log only, and this occurred at 8 hours post-treatment (p≤0.05). In contrast, transcripts for the anti-inflammatory cytokine TGF-β were only up-regulated at 1 hour post-low dose LPS treatment (p≤0.05), while being down-regulated at 3 (p≤0.01) and 8 hours (p≤0.05) post-LPS treatment (p≤0.01), and at 8 hours post-CpG ODN treatment (p≤0.05) (Figure 2D).

### Thrombocytes Up-regulate Transcripts for Anti-viral Cytokines and those Associated with Antigen Presentation in Response to TLR Ligands

**Figure 3:**
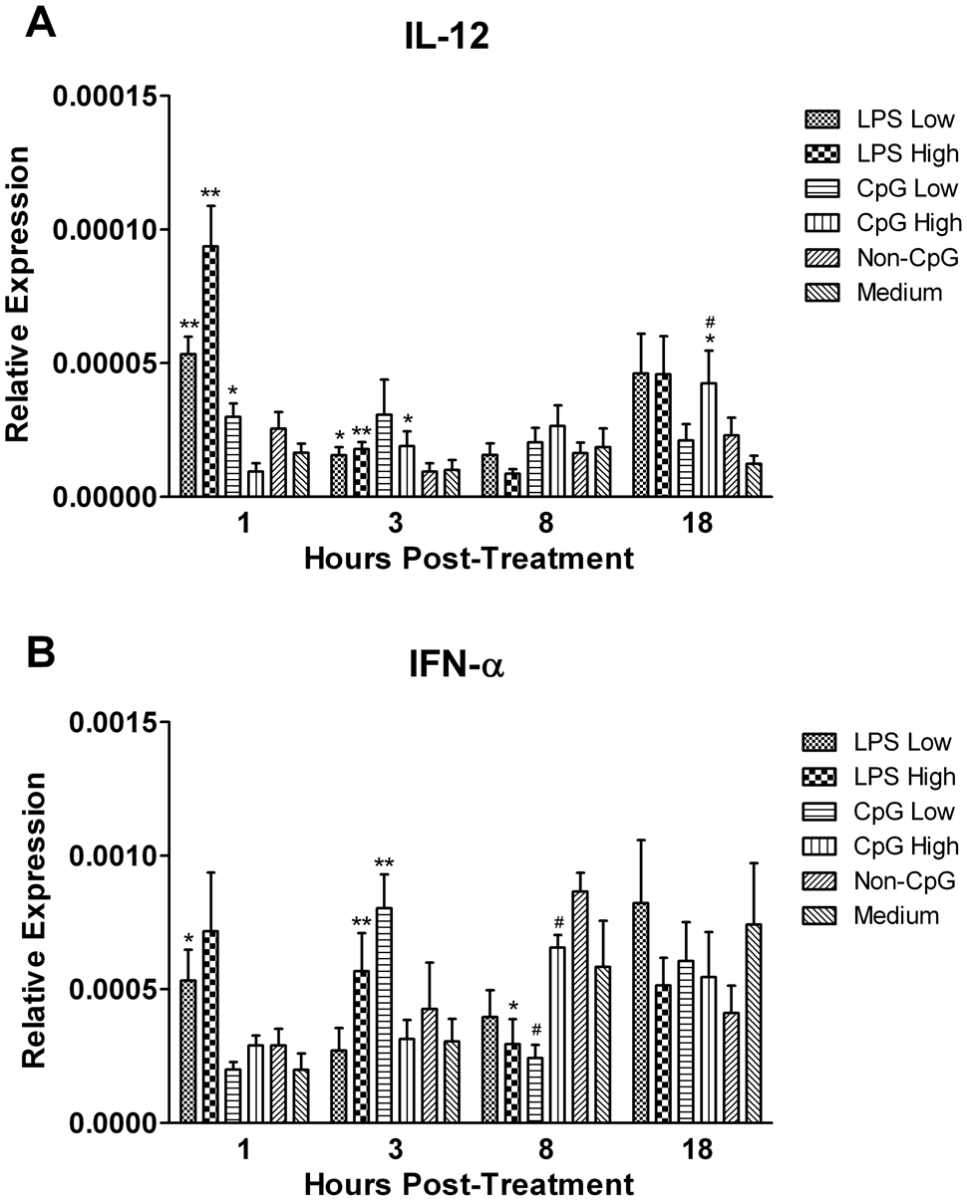
Relative gene expression of cytokines associated with anti-viral and T_H_1 responses. Relative gene expression of the cytokines IL-12 and IFN-α in chicken thrombocytes at 1, 3, 8 and 18 hours post-treatment with low (1 µg/mL) and high (5 µg/mL) doses of the TLR ligands LPS and CpG ODN. Data represent mean expression levels of target genes in 5 biological replicates relative to the house keeping gene β-actin ± standard error. Statistical significance between treatment groups and the medium control group was calculated using a paired student’s t test and was considered statistically significant if p≤0.05 (*) and p≤0.01 (**), and in the case of CpG ODN, considered statistically significant from the non-CpG ODN control group if p≤0.05 (#).

To determine if TLR ligands enhance the production of cytokines that may promote an anti-viral state, we quantified transcripts for IL-12 and IFN-α (Figure 3). Both LPS and CpG ODN induced the up-regulation of IL-12, with transcripts peaking at 1 hour (p≤0.01) and 18 hours (p≤0.05) post-treatment, respectively (Figure 3A). With respect to IFN-α, transcripts were significantly up-regulated in response to LPS at 1 (p≤0.05) and 3 hours (p≤0.01) post-treatment, while subsequently down-regulated at 8 hours (p≤0.01) post-treatment (Figure 3B). This was in contrast to CpG ODN treatment, as transcripts were only significantly up-regulated at 3 hours post-treatment with the low dose (p≤0.01) (Figure 3B). Despite up-regulation of IFN-α, IFN-β was not significantly up-regulated except for the LPS high and CpG low groups at 3 hours, which approached statistical significance (data not shown).

**Figure 4:**
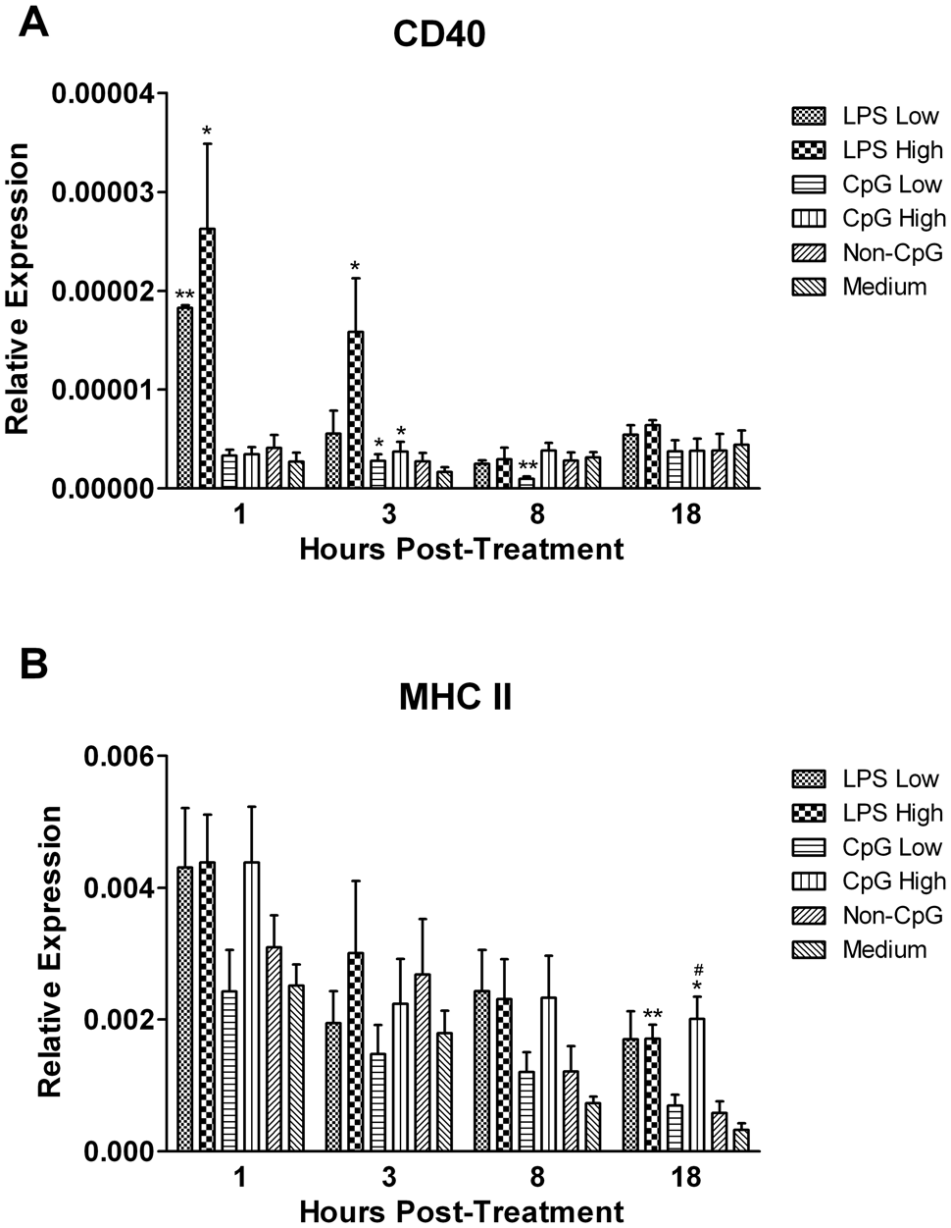
Relative gene expression of surface molecules. Relative gene expression of CD40 and MHC-II in chicken thrombocytes at 1, 3, 8 and 18 hours post-treatment with low (1 µg/mL) and high (5 µg/mL) doses of the TLR ligands LPS and CpG ODN. Data represent mean expression levels of target genes in 5 biological replicates relative to the house keeping gene β-actin ± standard error. Statistical significance between treatment groups and the medium control group was calculated using a paired student’s t test and was considered statistically significant if p≤0.05 (*) and p≤0.01 (**), and in the case of CpG ODN, considered statistically significant from the non-CpG ODN control group if p≤0.05 (#).

To gain some insight into any potential antigen presenting capabilities of thrombocytes, we quantified transcripts for both CD40 and major histocompatibility complex (MHC) class II (Figure 4). Although treatment with LPS resulted in a large (∼10 fold) up-regulation of CD40 transcripts at 1 (p≤0.05) and 3 hours (p≤0.05) post-treatment, transcripts were slightly up-regulated in response to CpG ODN at 3 hours post-treatment (p≤0.05), and subsequently down-regulated at 8 hours (p≤0.01) post-treatment (Figure 4A). However in contrast, CpG ODN induced an approximately 5-fold up-regulation in MHC-II transcripts at 18 hours-post treatment (p≤0.05), similar to that of LPS (p≤0.01) (Figure 4B).

### LPS, but not CpG ODN, Enhances Nitric Oxide Production and Phagocytic Activity of Thrombocytes

**Figure 5:**
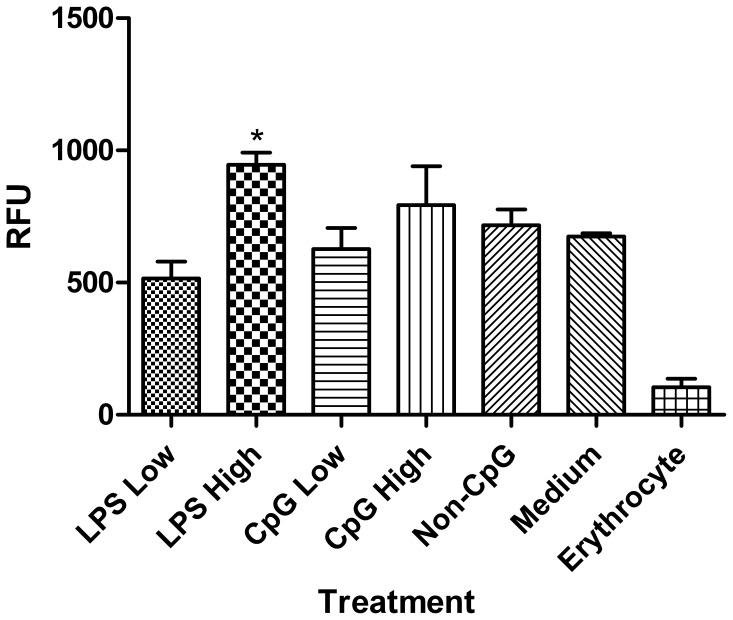
Assessment of phagocytosis. Assessment of phagocytosis in chicken thrombocytes at 2 hours post-treatment with low (1 µg/mL) and high (5 µg/mL) doses of the TLR ligands LPS and CpG ODN. Freshly isolated chicken erythrocytes were used as a non-phagocytic cell type control. Data represent mean relative fluorescence units (RFU) from 4 biological replicates ± standard error. Statistical significance between treatment groups and the medium control group was calculated using a paired student’s t test and was considered statistically significant if p≤0.05 (*).

**Figure 6:**
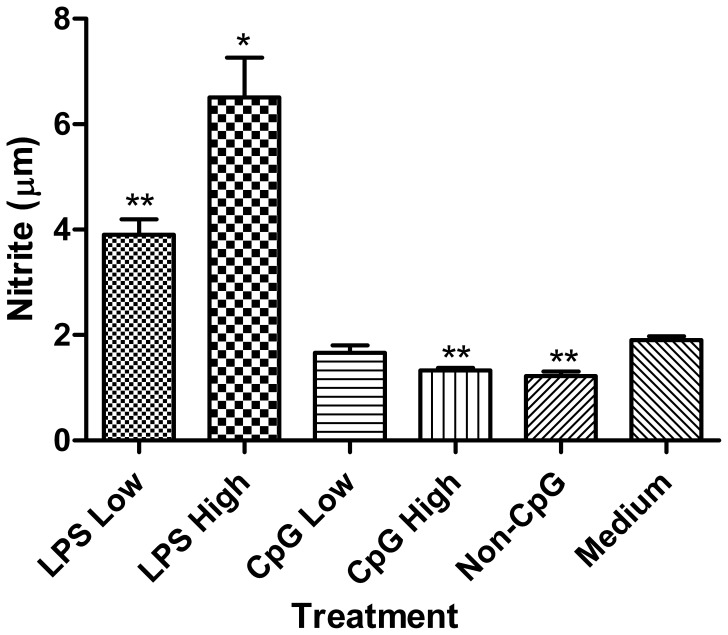
Assessment of nitrite production. Nitrite production in chicken thrombocytes at 48 hours post-treatment with low (1 µg/mL) and high (5 µg/mL) doses of the TLR ligands LPS and CpG ODN. Data represent mean nitrite quantity (µm) in culture supernatants from 4 biological replicates ± standard error. Statistical significance between treatment groups and the medium control group was calculated using a paired student’s t test and was considered statistically significant if p≤0.05 (*) and p≤0.01 (**).

In addition to cytokine production, it has been established that thrombocytes demonstrate other effector functions, including phagocytosis and nitric oxide production [4], [16] **.** Therefore, we hypothesized that these effector functions would be enhanced by TLR ligands. We found that LPS, but not CpG ODN, enhances the phagocytic uptake of fluorescent latex beads (p≤0.05) (Figure 5). Furthermore, although both doses of LPS enhanced (p≤0.05) the production of nitrite (which is used as surrogate marker for nitric oxide), treatment with the high dose of CpG ODN and non-CpG ODN resulted in a decrease (p≤0.01) in nitrite (Figure 6). In addition to CpG and LPS, we stimulated thrombocytes with ligands for TLRs 2, 3, 5 and 7 as well, and of these ligands, we found that only the TLR7 ligand R848 significantly up-regulated nitrite production (unpublished results).

## Discussion

In addition to their hemostatic effects, mammalian platelets contribute to aspects of host immunity by secreting several different cytokines that modulate the inflammatory response. Here, we report that TLR-stimulated thrombocytes may also contribute to immune responses by up-regulating pro-inflammatory and anti-viral genes, as well as genes associated with antigen presentation. Furthermore, TLR stimulation led to enhanced nitric oxide production and phagocytic activity of thrombocytes.

Platelets store their cytokines in granules which are released following encounters with pathogens, and this process is mediated in part by TLR stimulation [9]. As such, platelets express several different TLRs, a finding that, based on the results of the present study, may be extended to chicken thrombocytes as well. Our results suggest that thrombocytes constitutively express transcripts for TLRs 2, 3, 4, 5, 7 and 21, with transcripts for TLRs 2, 3 and 4 being highly expressed. Moreover, we found that thrombocytes respond to ligands for TLRs 4, 7 and 21, but not to ligands for TLRs 2, 3 and 5, which raises the possibility that these latter TLRs have low protein expression or may not be fully functional in this cell subset. Nevertheless, the potential for a single cell subset to express such a diverse repertoire of TLR transcripts is not unique to thrombocytes, as at least 6 different TLRs are constitutively expressed in chicken macrophages (HD11 cells), B cells and T cells, among others [17]. Together, these results show that thrombocytes may have the potential to respond to both viral and bacterial pathogens.

In mammals, many of the cytokines that are released by platelets contribute to the modulation of inflammatory responses [6]. Indeed, thrombocytes may also contribute to inflammatory responses as well, as they have previously been shown to express the pro-inflammatory cytokines IL-1β and IL-6 [5], [12]. Here, we report that thrombocytes also express IL-8 at the transcript level. Importantly, the magnitude of the response must be considered, as TLR ligands induced nearly 100 fold up-regulation of these pro-inflammatory cytokines from 1 hour through 18 hours post-treatment. When stimulated with similar doses of CpG ODN or LPS, other chicken cell subsets do not produce nearly as much. For example, chicken macrophages up-regulate IL-1β transcripts approximately 3 fold at 3 hours post-CpG ODN treatment, [18], while LPS up-regulates IL-1β and IL-8 transcripts 6–8 fold in chicken heterophils at 1 hour post-treatment [19]. Taken together, this may suggest an integral role of thrombocytes in initiating and sustaining inflammatory responses to bacterial and viral pathogens by rapidly and robustly up-regulating pro-inflammatory cytokines. In addition to the pro-inflammatory cytokines, we also demonstrate that thrombocytes express the anti-inflammatory cytokines TGF-β and IL-10 as well. This raises the possibility that thrombocytes may play a dual role in modulating the inflammatory response. In mammals, platelets also produce TGF-β, however the factors surrounding its release have not been thoroughly elucidated [6]. Interestingly, it appears that platelet-derived TGF-β may be important for the development of regulatory T cells (T_REG_), as individuals suffering from thrombocytopenia display decreased numbers of T_REG_, which are subsequently restored in response to treatments that increase platelet counts [20]. As chicken thrombocytes produce both TGF-β and IL-10, it is possible that thrombocytes fulfill a similar niche in chickens. Future research should determine both the factors that promote the up-regulation of TGF-β, and the biological significance of thrombocyte-derived TGF-β and IL-10 *in vivo* in regulating immune responses.

In addition to producing inflammatory cytokines, thrombocytes have previously been shown to produce IL-12, which helps to bias the immune response towards a T-helper (T_H_) 1 phenotype [5]. There was an up-regulation of IL-12 in thrombocytes response to LPS and CpG ODN, which raises the possibility that thrombocytes may be one of the cell subsets that contribute to the T_H_1-like responses observed *in vivo* following administration of LPS and CpG [21]. Interestingly, it has recently been proposed that highly pathogenic avian influenza virus (AIV) has the potential to replicate inside chicken thrombocytes and promote the up-regulation of some interferon inducible genes [15], which may suggest thrombocytes as one of the cell subsets contributing to the pro-inflammatory response observed following AIV infection [22]. In support of this notion, we found that thrombocytes express transcripts for type I interferons (IFN-α and β), as well as the interferon inducible gene 2′–5′ OAS, which all are involved in anti-viral responses, including responses against AIV [23], [24] **.** Furthermore, we found that thrombocytes up-regulate IFN-α in response to treatment with LPS, which is similar to what we observed *in vivo* in the spleens of chickens treated with LPS [21]. Interestingly, in the present experiment we observed a significant up-regulation of IFN-α in response to CpG ODN. Generally, CpG ODN can be divided into three classes (A, B and C) depending on their sequence of the CpG motifs [25]. In mammals, Class A ODNs are highly immunostimulatory to plasmacytoid dendritic cells (pDC) and thereby induce the production of IFN-α [26]. In contrast, class B ODNs weakly induce IFN-α production, but are highly immunostimulatory for B cells thereby resulting in enhanced IFN-γ production [26]. Lastly, class C ODNs are a mixture of Class A and B motifs, thereby moderately stimulating both pDCs and B cells. Since in the present work we observed a significant up-regulation of IFN-α in response to a class B CpG ODN, this raises the possibility that TLR21 might not share a similar motif-specific response with mammalian TLR9 and, thus, should be the subject of further investigation.

In addition to the professional antigen presenting cells (APCs), there are several other cell subsets that may occasionally present via MHC-II, such as gamma delta T cells [27]. Although it has previously been shown that chicken thrombocytes express the MHC-encoded B-G antigen complex as well as the co-stimulatory molecule CD40, it remained to be elucidated as to whether they expressed MHC-II and other related molecules [28], [29]. Here, we report that thrombocytes express transcripts for MHC-II as well as for the co-stimulatory molecule CD80, which raises the possibility that thrombocytes may serve as non-professional APCs. In support of this notion, we found that transcripts for MHC-II and CD40 were significantly up-regulated in response to LPS and CpG ODN, thereby resembling the TLR-mediated responses observed in other APCs, such as in chicken dendritic cells [30] and in chicken B cells (St. Paul et al, unpublished data). Based on our findings, future studies should be aimed at assessing whether thrombocytes demonstrate any antigen-presenting capabilities.

It has been proposed that thrombocytes are the primary circulating phagocyte in chickens [31]. Thrombocytes have previously been shown to phagocytose a variety of bacterial and viral pathogens, such as *Staphylococcus aureus* and *Salmonella enterica* serovar Typhimurium [16]. Moreover, it was reported that thrombocytes significantly increase their nitric oxide production in response to treatment with various strains of bacteria, which may be a result of TLR-mediated signaling [16]. In support of this, in the present study we found that LPS significantly enhanced both phagocytosis and nitric oxide production. Furthermore, our results suggest that thrombocytes are actively phagocytic, even without any TLR ligand treatments, as there was a nearly 5-fold increase in phagocytosis compared to the non-phagocytic cell type control. Nonetheless, the ability for TLR-ligands such as LPS to enhance phagocytosis and respiratory burst capabilities has been well studied in other phagocytes, such as in monocytes, macrophages and heterophils [32]–[34]. In contrast CpG ODN failed to enhance the phagocytic activity of thrombocytes and, in fact, decreased their production of nitric oxide. Although the reason behind this decrease is not known, it may be attributed to TLR-independent mechanisms, as we observed a similar phenomenon in the non-CpG ODN treated group. In mice, non-CpG ODNs have been shown to modulate the inflammatory response in a TLR9-independent manner [35], which may be mediated by certain intracellular DNA receptors [36]. In this regard, future studies may be aimed at elucidating the receptors and signaling pathways involved in responses to non-CpG ODN in chickens.

In conclusion, we demonstrate that thrombocytes constitutively express transcripts for several TLRs in addition to genes associated with inflammation, antigen presentation and anti-viral responses. Moreover, treatment with the TLR ligands LPS and CpG ODN significantly up-regulated many of these genes, while only LPS enhanced their nitric oxide production and phagocytic capabilities. Future studies may be aimed at investigating the contributions of thrombocytes to host immunity both *in vitro* and *in vivo*.
